# Unveiling a new sequence type in high-risk clonal complexes of virulent carbapenem-resistant *Pseudomonas aeruginosa* carrying *bla*_KPC−2,_ *bla*_NDM_ and *bla*_oxa−48_

**DOI:** 10.1007/s42770-025-01849-w

**Published:** 2026-02-18

**Authors:** Vitelhe Ferreira de Almeida, Vinicius Lopes Dias, Teresiama Velikkakam, Sabrina Royer, Elias Rodrigues de Almeida-Junior, Caio Augusto Martins Aires, André Oliveira Mota Junior, Isabella Macário Ferro Cavalcanti, Maria Amélia Vieira Maciel, Cristiane Silveira Brito, Rosineide Marques Ribas

**Affiliations:** 1 Laboratory of Molecular Microbiology, Federal University of Uberlandia, Uberlandia, Minas Gerais Brazil; 2 Department of Health Sciences, Federal University of the Semi-Arid Region – UFERSA, Mossoró, Brazil; 3 Laboratory of Microbiology and Immunology, Academic Center of Vitória (CAV), Federal University of Pernambuco (UFPE), Institute Keizo Asami (iLIKA- UFPE), Vitória de Santo Antão, Pernambuco, Brazil; 4 Medical Science Center, Federal University of Pernambuco, Recife, PE Brazil; 5 Department of Microbiology, Universidade Federal de Uberlândia (UFU), Uberlândia, Brazil; 6 Laboratory of Molecular Microbiology, Federal University of Uberlândia, Uberlândia, Minas Gerais Brazil

**Keywords:** High-risk clones, *P. aeruginosa*, Dissemination, Efflux pump genes, Clonal complex CC244

## Abstract

Brazil is a country with an approximate 50% prevalence rate of carbapenem-resistant *Pseudomonas aeruginosa* (CR-PA). Therefore, monitoring the spread of high-risk clones from complex genotypes of CR-PA isolates is crucial for developing effective strategies to control antimicrobial resistance. In the present study, we aimed to evaluate the dissemination of high-risk clones *P. aeruginosa* carrying *bla*_KPC-2_ in Brazil. In this study, 87 CR-PA isolates were analyzed for virulence (*exo*U, *exo*T, *exo*S, and *exo*Y) and resistance (*bla*_KPC_, *bla*_OXA-48_, *bla*_PDC_, *bla*_NDM_, *qnr*A, and *qnr*B) markers, detected by PCR and further assessed by RT-qPCR for *mex*A, *mex*B, *mex*E, *mex*X, *bla*_PDC_, *opr*D, and *amp*C genes. Clonal relatedness of the isolates was evaluated using multilocus sequence typing (MLST) and Pulsed-Field Gel Electrophoresis. Most isolates were obtained from tracheal secretions (65%). Among the resistance genes evaluated, the prevalent was *bla*_PDC-5_ (88%). Additionally, these strains were found carrying important genes, including *bla*_KPC-2_ (12%) and *bla*_OXA-48_ (4%). No overexpression of efflux pump genes or the *bla*_PDC_ and *amp*C genes was observed compared to the control strain (PAO1). Similarly, there was no reduction in *opr*D gene expression. The MLST analysis revealed that all evaluated strains belonged to novel sequence types (STs). These STs were associated with five different clonal complexes: ST5037, ST5040, ST5043 (CC244), ST5038 (CC155), ST5039 (CC235), ST5041 (CC277), ST5042 (CC639), and ST5044 (CC27). Eight new STs were identified in KPC-*P. aeruginosa* in Brazil. This finding suggests the need for consistent monitoring of KPC-producing *P. aeruginosa* to control the spread of high-risk clones in hospital settings.

## Introduction

In addition to *Pseudomonas aeruginosa* being placed in the category of ‘high priority’ of the World Health Organization’s (WHO) list of bacterial pathogens is regarded as one of the most difficult pathogens to manage in healthcare environments, primarily because it possesses inherent resistance to various classes of antimicrobials and has an exceptional ability to acquire additional resistance and spread rapidly [1, 4]. The outlook for managing infections caused by these microorganisms in hospitals, especially in low- and middle-income countries, is bleak.

Unfortunately, regarding hospital pathogens, the alarming global rise of *Klebsiella pneumoniae* carbapenemases (KPC) has threatened the effectiveness of last-resort antibiotics like carbapenems, significantly limiting therapeutic options. This is especially worrisome in the case of *P. aeruginosa* infections, as this species intrinsically exhibits multiple resistance mechanisms, such as low outer membrane permeability, active efflux systems, and the ability to form biofilms, which collectively reduce the efficacy of available antimicrobials and contribute to the persistence of severe and difficult-to-treat infections [4, 5].

Currently, several studies have provided evidence of the dissemination of high-risk international clonal complexes such as CC227, CC235 and CC245 of virulent *P. aeruginosa* strains carrying carbapenemases genes are highly capable of spreading within hospital environments and demonstrating prolonged persistence [6–8]. Through this study, we aim to gain valuable insights into the spread of *bla*_KPC_-carrying *P. aeruginosa* (KPC-*Pa*) clones in various public hospitals located in two distinct regions of Brazil, which can help strengthen infection control protocols in clinical settings.

## Methods

### Bacterial strain

The clinical *P. aeruginosa* strains were obtained from various public hospitals through collaborations with different research groups in two regions of Brazil (Southeast and Northeast), between January 2021 and December 2023. A total of eight hospitals were included in the investigation. All isolates were subjected to an initial screening for antimicrobial resistance and virulence genes, and those that met the inclusion criteria were selected for further molecular and genomic analyses. In addition to clinical isolates, three samples obtained from community water reservoirs were also included in this study to broaden the understanding of potential environmental reservoirs of *P. aeruginosa* carrying resistance determinants.

Strain identification was performed by the hospital laboratories using automated systems (VITEK II, bioMérieux, France) or Matrix-Assisted Laser Desorption Ionization–Time of Flight Mass Spectrometry (MALDI-TOF MS, Bruker Daltonics, Germany) or had been previously confirmed by the collaborating researchers. The isolates were subsequently sent to the Molecular Microbiology Laboratory at the Institute of Biomedical Sciences (ICBIM), Federal University of Uberlândia (Uberlândia, Minas Gerais, Brazil). They were inoculated on *Pseudomonas* Agar and BHI (Brain Heart Infusion) using the streak plate method to obtain pure cultures. They were stored in cryogenic tubes containing BHI broth added with 15% glycerol and stored at −80 °C for further for further molecular and genomic characterization.

### Polymerase chain reaction (PCR)

DNA extraction was carried out using a commercial NucleoSpin^®^ Tissue kit (MACHEREY-NAGEL), following the manufacturer’s instructions for bacterial samples. The presence of virulence genes from the type III secretion system (T3SS) (*exo*U, *exo*T, *exo*S, and *exo*Y genes) was assessed using endpoint PCR primer sequences as previously described [9]. The carbapenemases genes, *bla*_KPC_, *bla*_NDM_, *bla*_GIM_ and *bla*_OXA− 48_ were elucidated following the methodology by Poirel et al. [10]. And *bla*_PDC_ gene was investigated as reported by Ingti et al. [11]. The quinolone resistance genes *qnr*A and *qnr*B were also evaluated [12]. For each reaction, 1 µL of DNA, 10 µL of Milli-Q water, 0.75 µL (20 pmoles) of each primer, and 12.5 µL of GoTaq^®^ G2 colorless master Mix (Promega) were combined, resulting in a final reaction volume of 25 µL. The genes *bla*_KPC_ and *bla*_PDC_ were sequenced to confirm their presence of these amplicons. The genetic sequencing was performed by ACTGene Análises Moleculares LTDA, using 30 to 60 ng of purified DNA.

### Quantitative reverse transcription polymerase chain reaction (RT-qPCR)

Real-time quantitative PCR was used to measure the expression of the *mex*A, *mex*B, *mex*E, *mex*X, *bla*_PDC_, *opr*D and *amp*C genes using the primers listed in Supplementary Table 1. Twelve isolates of *P. aeruginosa* exhibiting a Crb-R/KPC^+^/PDC^+^ or Crb-R/KPC^−^/PDC^+^ (Crb-R = Carbapenem-resistant; KPC^**+**^= KPC-positive *Pseudomonas aeruginosa;* PDC^+^= PDC-positive *Pseudomonas aeruginosa*) profile were selected for this analysis (Supplementary Fig. 1). Additional criteria for strain selection included a higher prevalence of resistance genes and the origin of the samples.

The strain was quantified by real-time PCR using SYBR Green PCR Master Mix (Applied Biosystems) on a QuantStudio™ 3 Sequence Detection System (Applied Biosystems) as follows: denaturation at 95 °C for 30 s; then 40 cycles of 95 °C for fifteen seconds; and 60 °C for one minute. RNA and cDNA preparations were obtained using the PureLink^®^ RNA Mini Kit (Thermo Fisher Scientific) and the high-capacity cDNA Reverse Transcription Kit, respectively, according to the manufacturer’s instructions. The *rps*L gene was used as reference gene for normalizing the transcriptional levels of target genes. Data were compared to those obtained with the *rps*L gene using the threshold cycle (ΔΔCT) (relative) method, and the values ​​obtained were then normalized against the values ​​obtained for the susceptible isolate PAO1 (ATCC 15692). Each experiment was performed in triplicate in two independent assays.

The efflux pumps systems MexAB-OprM, MexEF-OprN, and MexXYwere considered overexpressed when the transcriptional levels of *mex*A, *mex*B, mexE and *mex*Xwere at least 2, 100, and 4-folds higher than those of the reference strain PAO1, respectively [13, 14]. Reduced *opr*D expression, overexpression of *amp*C and overexpression of *bla*_PDC_-like were considered relevant when their transcriptional levels were ≤ 70%, ≥ 10 and ≥ 10-folds respectively, compared to PAO1 strain [13, 14]. The transcriptional level analysis of each gene was performed using the unpaired, bi-directional Student’s t-test in Prism GraphPad version 8.0.1.

### Molecular typing

Strains of *P. aeruginosa* with *bla*_KPC_ genotypes were subjected to Pulsed-Field Gel Electrophoresis (PFGE). Genomic DNA from these isolates was digested using *Spe*I (Thermo Fisher Scientific), following the protocols outlined by Silva et al. [15]. The PFGE assay was performed using CHEF DRIII equipment (Bio-Rad, USA), in which the DNA fragments were separated on 1% (w/v) agarose gels in 0.5x TBE [Tris–borate–ethylene diamine tetra-acetic acid (EDTA)] buffer, using 6 V/cm, pulsed from 5 s to 90 s, for 20 h at 12 °C. The similarity of the PFGE patterns was assessed through computer comparison and interpreted using the GelAnalyzer 19.1 software. The clustering was performed using the unweighted-pair group method with average links, employing a 1% band and position tolerance. A similarity coefficient of 85% was selected to define clusters.

This study used Python (Python Software Foundation) to create a dendrogram. The process began with data preparation, which involved either a distance matrix or raw data points. Hierarchical clustering was performed using the `linkage` function from the `scipy.cluster.hierarchy` module. Finally, the dendrogram was generated and visualized with the `dendrogram` function from the same module, utilizing `matplotlib`. This approach enabled a clear and systematic visualization of the hierarchical relationships among the data points.

### The multilocus sequence typing (MLST)

The MLST of *P. aeruginosa* was conducted following the guidelines provided by the PubMLST database (https://pubmlst.org). Seven housekeeping genes— *acs*A, *aro*E, *gua*A, *mut*L, *nuo*D, *pps*A, and *trp*E—were amplified using specific primers recommended by PubMLST [16]. DNA extraction, reagent preparation, and amplification were performed using PCR according to the end-point PCR methodology. The resulting PCR products were purified using the Wizard^®^ SV Gel and PCR Clean-Up System - Rapid Protocol.

For sequencing, specific primers were used, as recommended by the PubMLST database, and the strains were sequenced by the ACTGenes Molecular Analyses company (Sanger sequencing). The obtained sequences were subsequently analyzed and compared to the allelic profiles in the PubMLST database to determine the isolates sequence types (STs). A diagram indicating the similarity between Sequence Types (STs) and Clonal Complexes (CCs) was constructed using the goeBURST algorithm and visualized with Phyloviz (PHYLOVIZ Online) software.

### Statistical analysis

Statistical analyses were performed using GraphPad Prism v.8.0.1 (GraphPad Software, San Diego, CA). Quantitative assays were compared using the Kruskal–Walli’s test, applying Dunn’s multiple comparison test. All tests were performed with a confidence level of 95% and statistical significance was defined as *P* < 0.05.

## Ethical approval

The research was approved by the Federal University of Uberlandia Committee of Ethics in Research with Human Participants (Approval No. 2.527.621).

## Results

**Fig. 1:**
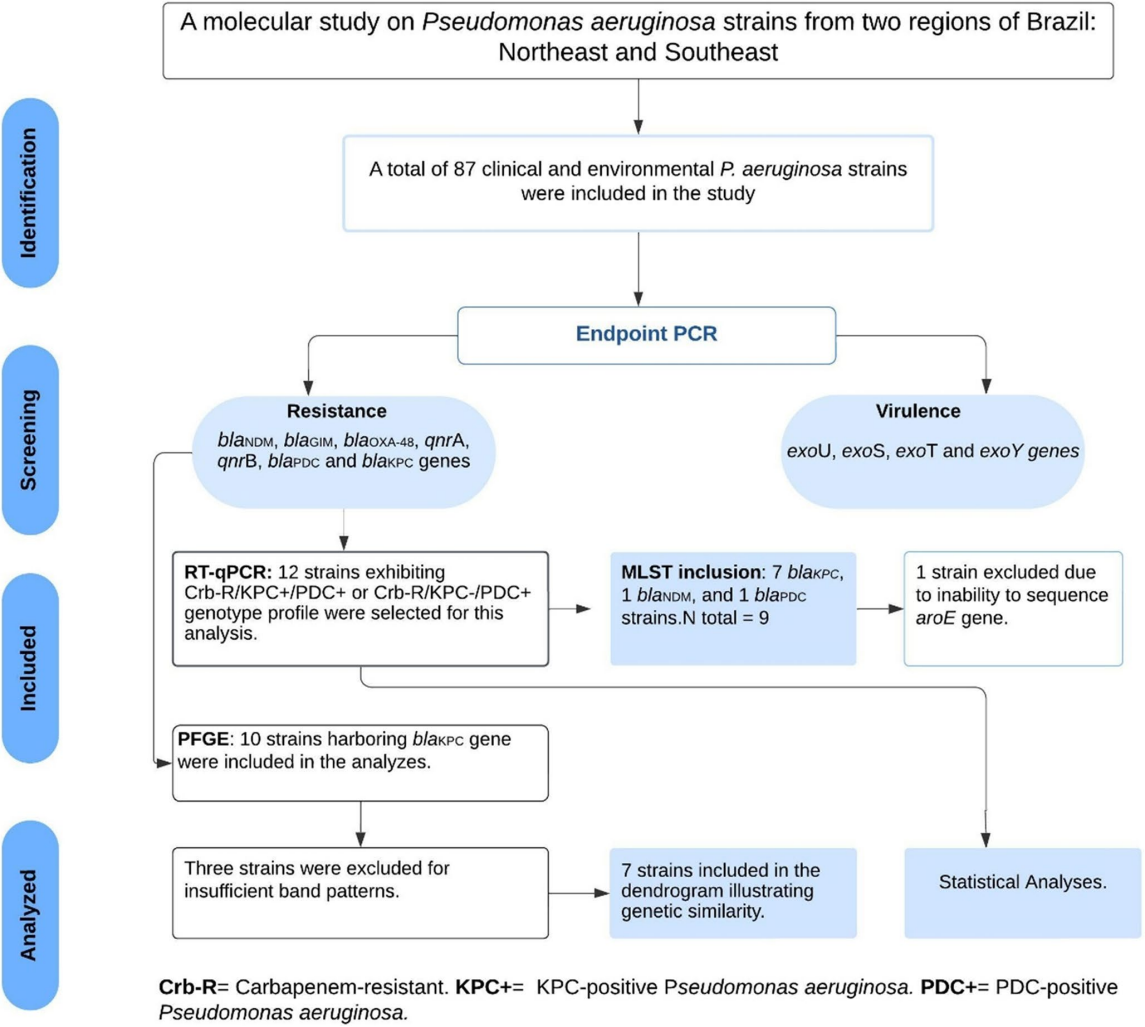
Flow diagram of study selection

From 2022 to 2023, a total of 87 isolates of *P. aeruginosa* obtained from various clinical and environmental sources were included in this study (Fig. 1). Most isolates were obtained from tracheal secretions 52/87 (65%), blood cultures 9/87 (10%) and urine cultures 7/87 (8%) (Table 1). The remaining isolates were primarily derived from other types of secretions (14%). Additionally, three samples included in this study were isolated from municipal drinking water reservoirs, in the southeastern region of Brazil.

**Table 1:** Molecular characterization of resistance and virulence genes in *Pseudomonas aeruginosa* strains from various sources evaluated in this study

Source	Total	Beta-lactam resistance-associated genes					Quinolone resistance-associated genes			Type III secretion system (T3SS)–associated genes			
		***bla***_**KPC−2**_	***bla***_**PDC−5**_	***bla***_**NDM**_	***bla***_**OXA−48**_		***qnr*A**	***qnrB***		***exo*U**	***exo*S**	***exo*T**	***exo*Y**
Tracheal secretion	57 (65.5)	6 (10.5)	53 (92.9)	1 (1.7)	0 (0)		1 (1.7)	4 (7.0)		17 (29.8)	37 (64.9)	51 (89.4)	38 (66.6)
Urine	7 (8.0)	1 (14.2)	7 (100)	0 (0.0)	0 (0.0)		0 (0.0)	0 (0.0)		1 (14.2)	5 (71.4)	5 (71.4)	4 (57.1)
blood	9 (10.3)	0 (0.0)	7 (77.4)	0 (0.0)	0 (0.0)		0 (0.0)	0 (0.0)		3 (33.3)	5 (55.5)	7 (77.7)	6 (66.6)
Environmental	3 (3.4)	0 (0.0)	3 (100)	0 (0.0)	2 (66.6)		0 (0.0)	0 (0.0)		2 (66.6)	3 (100)	3 (100)	3 (100)
Others*	12 (13.7)	3 (23.0)	6 (50.0)	0 (0.0)	1 (7.6)		0 (0.0)	0 (0.0)		5 (41.6)	8 (66.6)	9 (75.0)	7 (63.6)
**Total**	**87 (100)**	**10 (11.4)**	**77 (88.5)**	**1 (1.1)**	**3 (3.4)**		**1 (1.1)**	**4 (4.5)**		**28 (32.1)**	**58 (66.6)**	**75 (86.2)**	**58 (66.6)**

* Secretions from the ear, oral cavity, and wounds, as well as secretions from unknown sources. Synovial fluid, and samples provided without information on the site of infection

These samples are representative of two economically distinct major regions of the country: the Southeast and Northeast, encompassing four states—Minas Gerais (75 strain), São Paulo (3 strain [environmental isolates]), Alagoas (2 strain), Rio Grande do Norte (4 strain), and Pernambuco (7 strain).

Among the resistance genes evaluated, the *bla*_PDC-5_ gene showed the highest prevalence. Surprisingly, a significant proportion of *P. aeruginosa* strains carried *bla*_KPC−2_ (12%), and *bla*_OXA−48_ (4%). The 66% of the enviromental isolates carried *bla*_OXA−48_. Only one isolate carried the *bla*_NDM_ gene (1%). Overall, the strains did not show significant frequencies for quinolone resistance genes, with *qnr*B detected in 5% of the strains and *qnr*A in 1%. Additionally, a significant number of strains carried the *exo*U (32%) and *exo*S (66%) genes (Table 1).

Out of the 12 strains selected for the overexpression analysis of efflux pumps (*mex*A, *mex*B, *mex*E, *mex*X), 100% carried the *bla*_PDC_ gene and 66% harbored *bla*_KPC−2_. Furthermore, the expression of *bla*_PDC_, *amp*C, and porin (*opr*D) genes was analyzed using RT-qPCR. The characteristics of these strains are detailed in Supplementary Fig. 1. Among these strains, 50% harbored the *exo*U gene, while 75% harbored the *exo*S gene. No statistically significant overexpression of these efflux pump genes was observed compared to the control strain (PAO1) (Table 2; Supplementary Fig. 2). The same was observed for the *bla*_PDC_ and *amp*C genes. Regarding the evaluation of the *opr*D gene, no significant reduction was observed compared to the control (Table 2; Supplementary Fig. 3).

**Table 2:** Transcriptional analysis by RT-qPCR to measure the expression of the *mex*A, *mex*B, *mex*E, *mex*X, *bla*_PDC_, *opr*D and *amp*C genes

	*mex*A	*mex*B	*mex*E	*mex*X	*bla*PDC	*opr*D	*amp*C	Genotype
PA06H	0.3 ± 0.1	0.3 ± 0.3	1.4 ± 1.3	2.4 ± 0,03	0.6 ± 0.63	0.5 ± 0.3	0.03 ± 0.04	*bla*_KPC_^+^*bla*_PDC_+
PA11H	0.4 ± 0.2	0.3 ± 0.2	1.2 ± 0.8	0.68 ± 0.9	0.7 ± 0.4	5.3 ± 2.7	0.03 ± 0.04	*bla*_KPC_+*bla*_PDC_+
PA14H	0.2 ± 0.1	0.1 ± 0.09	0.1 ± 0.1	0.2 ± 0.2	0.1 ± 0.1	0.2 ± 0.1	0.01 ± 0.02	*bla*_KPC+_*bla*_PDC_+
PA01M	0.5 ± 0.3	0.1 ± 0.09	2.2 ± 1.4	0.06 ± 0.1	0.8 ± 0.3	0.2 ± 0.07	0.05 ± 0.08	*bla*KPC + *bla*+
PA04M	0.5 ± 0.3	0.2 ± 0.1	0.8 ± 0.3	1.6 ± 1.4	0.4 ± 0.2	0.8 ± 0.5	0.2 ± 0.3	*bla*_PDC_+
PA03U	0.6 ± 0.2	0.09 ± 0.08	0.7 ± 0.3	1.9 ± 1.6	0.3 ± 0.2	1.3 ± 0.6	2.0 ± 2.2	*bla*_PDC_+
PA11U	0.6 ± 0.2	1.0 ± 1.3	1.4 ± 0.6	2.4 ± 1.8	0.6 ± 0.7	1.4 ± 0.7	0.5 ± 0.8	*bla*_PDC_+
PA19U	1.6 ± 1.0	0.4 ± 0.16	1.5 ± 1.0	2.3 ± 3.4	0.6 ± 0.7	1.0 ± 0.4	0.2 ± 0.2	*bla*_PDC_+
PA07R	0.9 ± 0.6	1.6 ± 3.0	1.7 ± 1.4	3.6 ± 3.3	0.7 ± 0.5	1.8 ± 0.9	1.7 ± 2.4	*bla*_KPC_+*bla*_PDC_+
PA08R	0.5 ± 0.1	0.4 ± 0.2	1.5 ± 0.9	6.6 ± 4.4	1.0 ± 0.9	0.6 ± 0.3	0.2 ± 0.3	*bla*_KPC_+*bla*_PDC_+
PA10R	0.6 ± 0.4	0.4 ± 0.4	1.5 ± 0.8	2.3 ± 2.5	0.7 ± 0.2	0.8 ± 0.5	3.6 ± 4.3	*bla*_KPC_+*bla*_PDC_+
PA24 J	0.2 ± 0.1	0.2 ± 0.1	0.8 ± 0.6	3.2 ± 3.9	0.7 ± 0.3	1.3 ± 0.9	0.001 ± 0.004	*bla*_PDC_^+^*qnrB*^*+*^
**ATCC PAO1**	**1.1 ± 0.5**	**1.4 ± 1.1**	**1.2 ± 0.7**	**1.9 ± 1.7**	**1.2 ± 0.7**	**1.0 ± 0.3**	**3.9 ± 7.8**	**-**

**Fig. 2:**
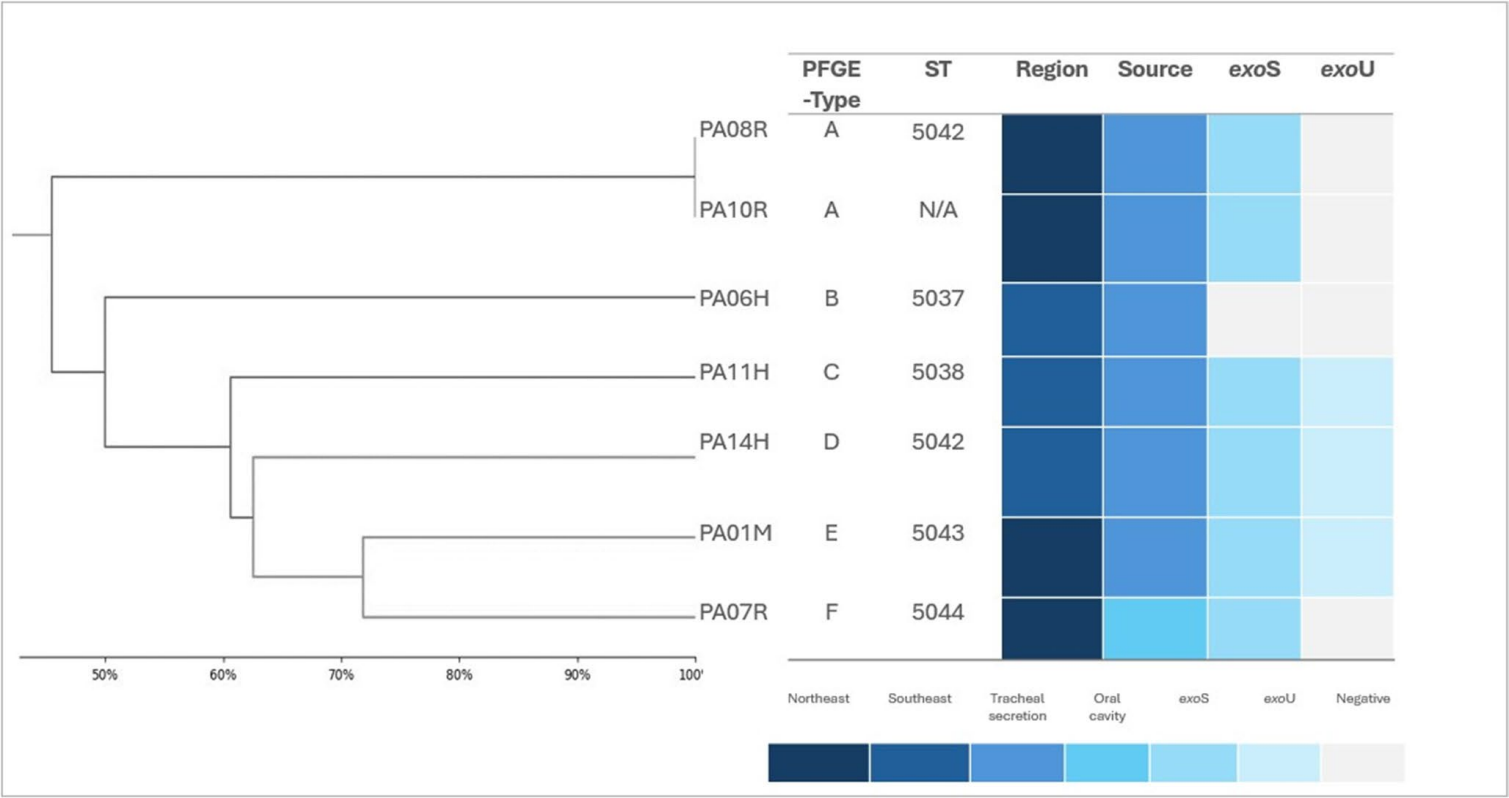
A dendrogram representing the pulsed-field gel electrophoresis analysis, pulsotypes, lineages, genotypes, sequence type (ST), and source in carbapenem-positive *P. aeruginosa* strains carrying *bla*_KPC−2_ and *bla*_PDC−5_ genes. Three isolates (PA12H; PA46; PA67R) did not produce sufficient bands in two separate experiments, and therefore their results are not included. Sequencing of the *aro*E gene for the PA10R strain was unsuccessful, leading to its exclusion from the final MLST results

The molecular typing analysis using PFGE was conducted on all isolates containing *bla*_KPC−2_ gene, resulting in six pulsotypes (A-F) identified. Clone A included two isolates, originating from the same northeastern region of the country. Interestingly, 30% of these strains were *exo*U^+^ and 60% were *exo*S^+^ (Fig. 2). In two attempts, three isolates failed to generate sufficient band patterns in two independent experiments, leading to their exclusion from the analysis. A flow diagram of study selections is shown in Fig. 1.

**Fig. 3:**
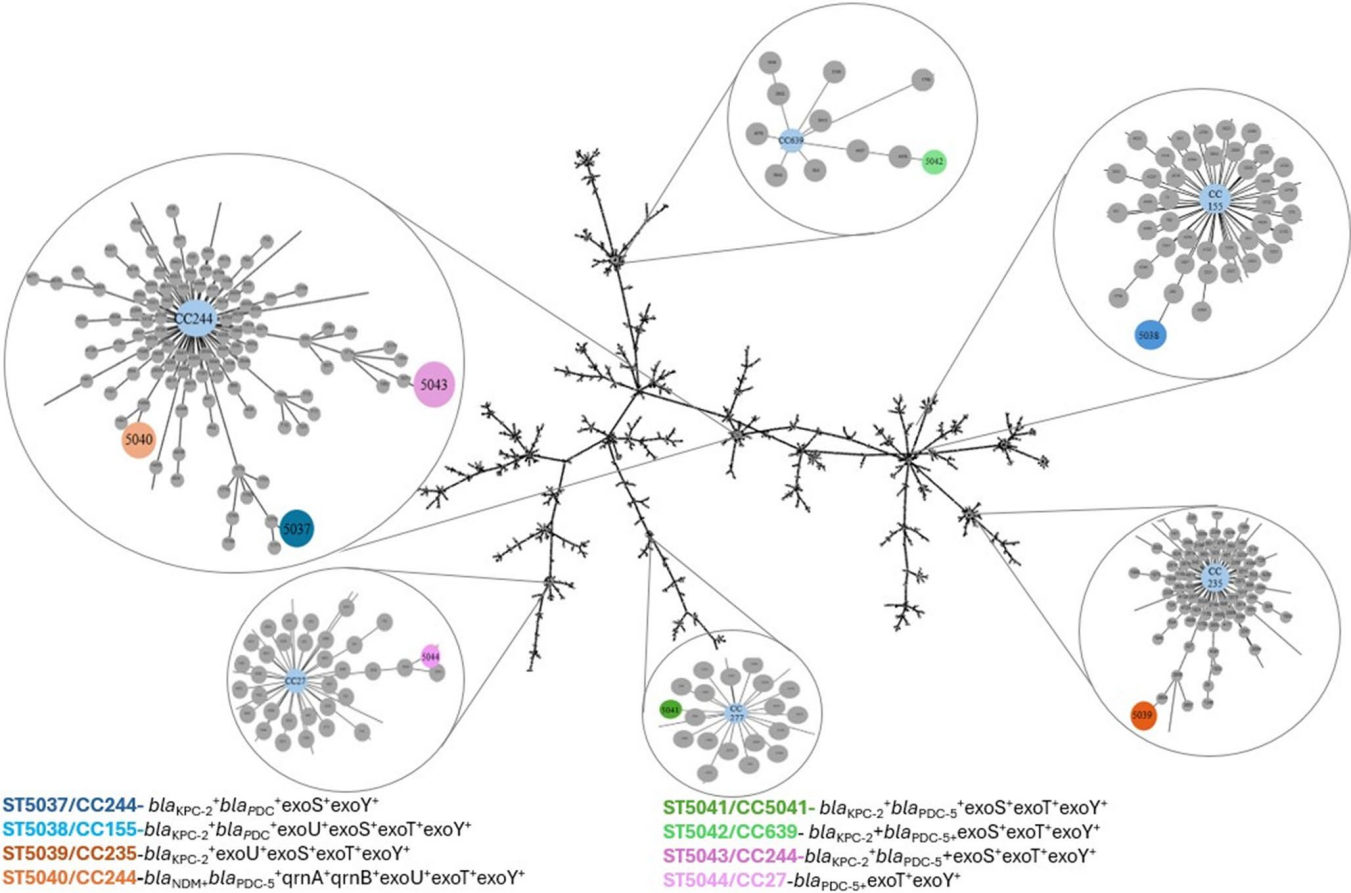
Diagram constructed using the goeBURST algorithm and visualized with the Phyloviz software (PHYLOVIZ Online), indicating the similarity among Sequence Types (STs). The Clonal Complexes (CCs) and STs observed in the present study are magnified and highlighted by color. The clonal complexes are shown in blue, while the different STs are represented by various colors

Among the strains analyzed by RT-qPCR, eight were selected for MLST analysis (Supplementary Fig. 1, Fig. 1). The MLST analysis revealed that all evaluated strains belonged to novel sequence types (STs), none of which had been previously reported in the literature. These STs were associated with five different clonal complexes (CC): CC244 (ST5037, ST5040, ST5043), CC155 (ST5038), CC235 (ST5039), CC277 (ST5041), CC639 (ST5042), and CC27 (ST5044) (Fig. 3). Notably, three of these strains belong to the high-risk clonal complex CC244 (PA06H, PA23H, PA01M), originating from the two macro-regions we evaluated. Sequencing of the *aro*E gene for one of the strains was unsuccessful, leading to its exclusion from the final MLST results.

## Discussion

According to the most recent data from the Antimicrobial Testing Leadership and Surveillance program [17], carbapenem resistance in *P. aeruginosa* clinical strains remain highest in the Middle East, with countries like Saudi Arabia and Kuwait exhibiting elevated resistance rates. South America, particularly Brazil and Colombia, also continues to experience significant increases in carbapenem resistance ranging from 43% to 60% in Brazil and 14% to 40% in Colombia [4, 17–20]. Already the Europe and North America follow with lower, yet concerning, resistance levels, especially in intensive care units (ICUs) [21, 22]. The persistence and spread of carbapenemases genes such as *bla*_KPC_ and *bla*_VIM_, alongside resistance mechanisms like *opr*D porin repression and efflux pumps overexpression, are fueling these resistance patterns [17, 20, 22].

Contrary to what was expected, we did not observe hyperexpression of the evaluated efflux pumps, *bla*_PDC_ gene or repression of the *opr*D gene in this study. Our investigation suggests that in the evaluated strains, resistance was primarily associated with mobile genetic elements, probably plasmids, harboring resistance genes, including those encoding *bla*_KPC_, *bla*_NDM_ in clinical isolates, as well as *bla*_OXA−48_ in environmental isolates. These elements contribute to the dissemination of multidrug resistance to antibiotics, driving the rapid spread of resistance observed in the current scenario.

A Brazilian study conducted over seven years conducted by Kiffer et al. [4] demonstrated that in 2015, the presence of the *bla*_KPC_ gene in *P. aeruginosa* was only 2.5%, increasing to 9.6% to in 2017. Additionally, the study revealed an increase in the prevalence of the *bla*_NDM_ and a reduction of *bla*_SPM_ over time in *P. aeruginosa.* This indicates a change in the genotypic profile of these strains over time [4, 23]. Overall, the literature highlights a concerning trend in the global dissemination of *bla*_KPC_ genes, emphasizing the need for continued surveillance and research efforts to address this public health challenge [5, 6].

Further complicating this scenario, the strains studied here exhibited significant virulence associated with the T3SS. Strains showing a high-virulence genotype (e*xo*U), have been linked to worse clinical outcomes and higher mortality rates [24–26]. In this study, we observed that carbapenemases-positive *P. aeruginosa* strains showed higher frequencies of the *exo*U gene. Moreover, the literature demonstrates a correlation between multidrug resistance and *exo*U production, similarly evidenced in our findings, particularly concerning important resistance genes [24–29].

Although this study identified eight novel sequence types (STs), the surveillance also revealed five STs (ST5037/CC244, ST5040/CC244, ST5043/CC244, ST5039/CC235, ST5041/CC277) that belong to the top 10 globally disseminated clonal complexes in KPC-*Pa*, which are also among clonal complexes prevalent in Brazil (CC235, CC244, CC277) [6, 7]. Interestingly, all of these were associated with the same infection site category: tracheal secretion. The global clonal complex 235 is considered a high-risk epidemic clone with a global distribution and strong drug resistance [6–8]. This clone has a greater ability to acquire resistance genes, and this phenotype is more frequently observed compared to other strains [6, 30, 31].

Recent studies have highlighted the spread of other high-risk clones, in addition to CC235, which have a significant impact on the dissemination of multidrug-resistant genotypes, such as CC244. While CC244 is a highly prevalent clone not always linked to multidrug-resistant or extensively drug-resistant (MDR/XDR) profiles, it typically exhibits an *exo*S^+^/*exo*U^–^ genotype but carries significant resistance genes, such as *bla*_KPC−2_, as we have confirmed. Similarly, ST277 is highly prevalent in Brazil and is specifically associated with São Paulo Metallo-β-lactamase (SPM) and other metallo-β-lactamases [8, 32, 33]. Although we have not confirmed the presence of SPM in these strains, the presence of the clonal complex has been detected [8, 32].

Surprisingly, only two *bla*_KPC−_positive gene strains, isolated from tracheal secretions in the same region, were identified as clones by PFGE. This is particularly surprising because KPC-producing *P. aeruginosa* is often associated with clonal dissemination in hospital environments, facilitated by cross-contamination through healthcare professionals’ hands. The number of clonally related isolates suggests that diverse genetic backgrounds may be involved in the spread of *bla*_KPC_ in these regions. Given that this study characterizes novel sequence types (STs) harboring this gene. It is essential to investigate in greater detail how the associated genes identified in this study contribute to the successful dissemination of these clones in hospital settings. As observed in various high-risk international clones in different bacteria, such as *P. aeruginosa*, *Klebsiella pneumoniae*, and *Escherichia coli*, a clone equipped with a combination of virulence factors and a high level of resistance genes can pose a significant threat to public health, particularly in low- and middle-income countries like Brazil [8, 34–36]. This makes continuous surveillance crucial for the future.

## Conclusions

Eight new STs were identified in *P. aeruginosa* carrying the *bla*_KPC−2_ gene in Brazil, which are part of globally common clonal complexes and ranked among the top 10 high-risk clones. This observation highlights the critical need for comprehensive and continuous surveillance. The mechanisms of carbapenem resistance in *P. aeruginosa* are complex and currently involve the alarming presence of the *bla*_KPC−2_, *bla*_NDM_, and *bla*_OXA−48,_ as observed here. The data generated in this study provide critical information that will help reinforce global strategies for controlling the dissemination of these strains.
